# UNC93B1 Mediates Innate Inflammation and Antiviral Defense in the Liver during Acute Murine Cytomegalovirus Infection

**DOI:** 10.1371/journal.pone.0039161

**Published:** 2012-06-18

**Authors:** Meredith J. Crane, Pamela J. Gaddi, Thais P. Salazar-Mather

**Affiliations:** Division of Biology and Medicine, Department of Molecular Microbiology and Immunology, Brown University, Providence, Rhode Island, United States of America; National Institute of Allergy and Infectious Diseases – Rocky Mountain Laboratories, United States of America

## Abstract

Antiviral defense in the liver during acute infection with the hepatotropic virus murine cytomegalovirus (MCMV) involves complex cytokine and cellular interactions. However, the mechanism of viral sensing in the liver that promotes these cytokine and cellular responses has remained unclear. Studies here were undertaken to investigate the role of nucleic acid-sensing Toll-like receptors (TLRs) in initiating antiviral immunity in the liver during infection with MCMV. We examined the host response of UNC93B1 mutant mice, which do not signal properly through TLR3, TLR7 and TLR9, to acute MCMV infection to determine whether liver antiviral defense depends on signaling through these molecules. Infection of UNC93B1 mutant mice revealed reduced production of systemic and liver proinflammatory cytokines including IFN-α, IFN-γ, IL-12 and TNF-α when compared to wild-type. UNC93B1 deficiency also contributed to a transient hepatitis later in acute infection, evidenced by augmented liver pathology and elevated systemic alanine aminotransferase levels. Moreover, viral clearance was impaired in UNC93B1 mutant mice, despite intact virus-specific CD8+ T cell responses in the liver. Altogether, these results suggest a combined role for nucleic acid-sensing TLRs in promoting early liver antiviral defense during MCMV infection.

## Introduction

Initiation of inflammation following infection requires recognition of the invading microbe by innate immune pattern recognition receptors (PRRs) that signal in response to pathogen-associated molecular patterns (PAMPs). PRRs recognize self- and microbe-associated molecules [1]–[4]. Members of the Toll-like receptor (TLR) family of PRRs are transmembrane receptors that are expressed either on the cell surface or within the endosomal compartment and respond to a variety of PAMPs [1]. Murine TLR3, TLR7 and TLR9 are expressed in the endolysosome and are implicated in recognition of viral dsRNA, ssRNA and dsDNA, respectively [1], [5]–[11]. Ligation of the nucleic acid-sensing TLRs results in transcription of antiviral genes including type I IFNs (IFN-α/β) and proinflammatory cytokines [1]. TLR3 responses require signaling through the adaptor molecule Toll/IL-1R domain-containing adapter-inducing interferon-β (TRIF), while TLR7 and TLR9 are dependent on the adaptor molecule myeloid differentiation primary response gene 88 (MyD88) to activate transcription factors and induce gene transcription [1], [12]–[14].

Murine cytomegalovirus (MCMV) is a betaherpesvirus that can establish acute infection in multiple organs including the liver. Acute MCMV infection induces an early systemic proinflammatory cytokine response including high levels of type I IFNs, IFN-γ, IL-12 and TNF-α [15]–[19]. Infection in the liver induces early production of IFN-α, predominantly by plasmacytoid dendritic cells (pDCs), by 40 h post-infection [20], [21]. Type I IFN production mediates downstream responses including chemokine and cytokine production as well as monocyte/macrophage, natural killer (NK) cell and T cell recruitment [20]–[23]. Early type I IFN signaling is necessary for NK cell recruitment to the liver, where they deliver the antiviral cytokine IFN-γ within the first 48 h post-MCMV infection [23]. The NK cell IFN-γ response is an important early step in the control of liver infection [24], [25]. This response induces IFN-γ-dependent chemokines, which contribute to the recruitment of CD8+ T cells to the liver [26]. Liver CD8+ T cell responses occur by days 5 and 7 post-MCMV infection and are an important source of cytokines late in acute infection that contribute to resistance against MCMV [26]–[30].

While early responses to MCMV infection in the liver are well understood, it remains unclear how the virus is sensed in this compartment. This is in contrast with other sites, namely the spleen, in which studies by our group and others have definitively shown a role for TLR9 and MyD88 signaling in IFN-α, proinflammatory cytokine and cellular responses in addition to restriction of virus replication [6], [20], [31], [32]. Although TLR7 alone does not appear to have a strong role in MCMV recognition, TLR7 and TLR9 combined deficiency was shown to severely impair pDC responses against MCMV in the spleen [33]. A significant but minor role for TLR3 signaling in the spleen has also been suggested in response to MCMV infection [6]. In the liver, however, studies by our group have demonstrated that early innate responses are TLR9-independent but MyD88-dependent [20]. Liver pDCs from mice genetically deficient in TLR9 produce wild-type (WT) levels of IFN-α at 40 h post-MCMV infection, with intact downstream cellular and proinflammatory cytokine responses. Further, TLR9-deficient mice do not exhibit elevated liver viral titers. Conversely, MyD88-deficient mice have severely impaired liver cytokine and cellular responses, and are unable to control virus replication in this compartment [20], [32]. MyD88 is a common adaptor molecule for TLR9 and TLR7 signaling; however, evaluation of TLR7-deficient mice also demonstrated that TLR7 signals alone were not required to initiate liver antiviral defense [20].

These TLR-independent but MyD88-dependent antiviral responses suggested possible redundancies among TLR signals in the liver compartment in response to MCMV infection [20], [32]. To investigate this possibility, we utilized mice containing an H412R missense mutation in the endoplasmic reticulum protein UNC93B1 to address the combined function of nucleic acid-sensing TLRs in the liver during acute MCMV infection. The UNC93B1 mutation (known as ‘triple d’ or ‘3d’) impairs signaling through TLR3, TLR7 and TLR9 due to improper trafficking of these receptors to the endosomal compartment, and has been shown to affect exogenous antigen presentation [34], [35]. Our studies show that proinflammatory cytokine production after early infection with MCMV is dependent on UNC93B1. Further, UNC93B1 deficiency exacerbates liver disease and increases viral burden, although MCMV-specific CD8+ T cell responses are not impaired. Collectively, these results suggest a level of redundancy within the liver to promote viral recognition by demonstrating that a combination of nucleic acid-sensing TLRs contributes to innate inflammatory responses during MCMV infection.

## Results

### Systemic cytokine production is impaired in 3d mice during MCMV infection

**Figure 1:**
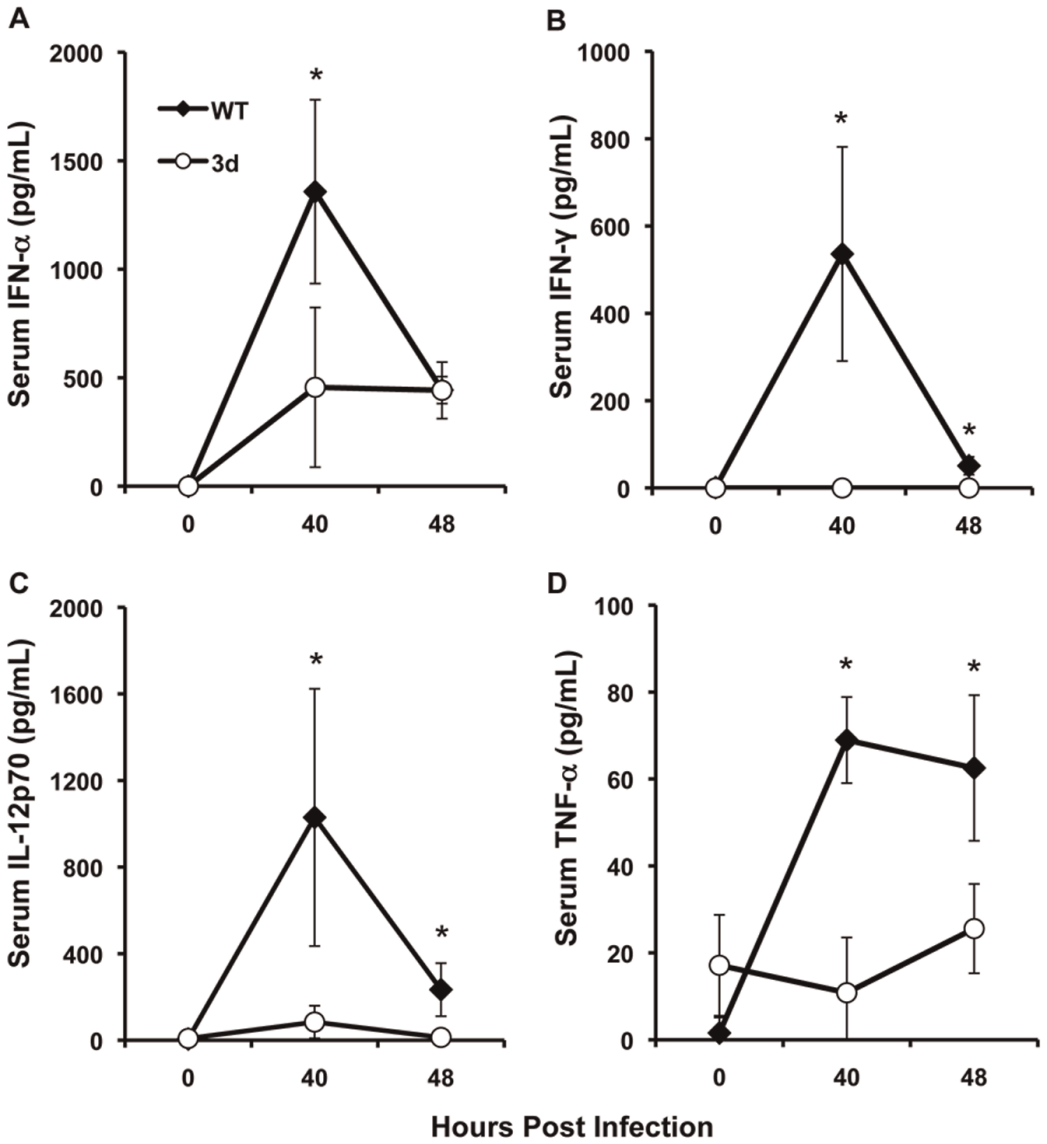
Effect of the 3d mutation on systemic cytokine production during MCMV infection. C57BL/6 (WT) or 3d mice were either uninfected or infected for 40 h or 48 h with MCMV. Serum was collected at indicated time points and tested for (A) IFN-α, (B) IFN-γ, (C) IL-12p70 and (D) TNF-α production by sandwich ELISA. Data are the combined results from two to three independent experiments and show the mean ± SD (n = 3–10 mice per group for each time point tested). Asterisks denote p values≤0.01.

Considering the potential of endosomal TLR signals to induce proinflammatory cytokine expression, UNC93B1 deficient 3d mice were first assessed for systemic IFN-α, IFN-γ, IL-12 and TNF-α production during early infection with a moderate (5×10^4^ PFU) dose of MCMV. C57BL/6 (WT) and 3d mice were uninfected or MCMV-infected for 40 h or 48 h. Serum was collected at indicated time points and IFN-α, IFN-γ, IL-12p70 and TNF-α were measured by enzyme-linked immunosorbent assay (ELISA). In WT mice, maximal production of IFN-α, IFN-γ and IL-12p70 was detected at 40 h post-MCMV infection before declining by 48 h post-infection (Fig. 1, A–C). In contrast, 3d mice exhibited lower serum levels of these cytokines in response to MCMV infection. Specifically, while serum IFN-α reached 1300±420 pg/mL at 40 h post-MCMV infection in WT mice, IFN-α production was reduced by three-fold at this infection time point in 3d mice (450±300 pg/mL), with comparable levels maintained at 48 h post-infection (Fig. 1A). Likewise, while average IFN-γ concentrations in WT mice reached maximal levels of 530±245 pg/mL at 40 h post-MCMV infection, 3d mice failed to induce detectable levels of this cytokine (Fig. 1B). IL-12p70 production similarly peaked at 40 h post-MCMV infection in WT mice, with levels reaching 1000±600 pg/mL. 3d mice, however, produced 12-fold less IL-12p70 at this infection time point (Fig. 1C). Serum TNF-α levels were elevated in response to MCMV infection at both 40 h and 48 h post-infection in WT mice (69±10 and 63±17 pg/mL, respectively, Fig. 1D). In 3d mice, average concentrations of TNF-α were 6-fold lower than WT at 40 h and 2.5-fold lower than WT at 48 h after infection. These results indicate a requirement for endosomal TLR signals for early systemic proinflammatory cytokine production, and concur with previous reports [35].

### Liver cytokine production is impaired in MCMV-infected 3d mice

**Figure 2:**
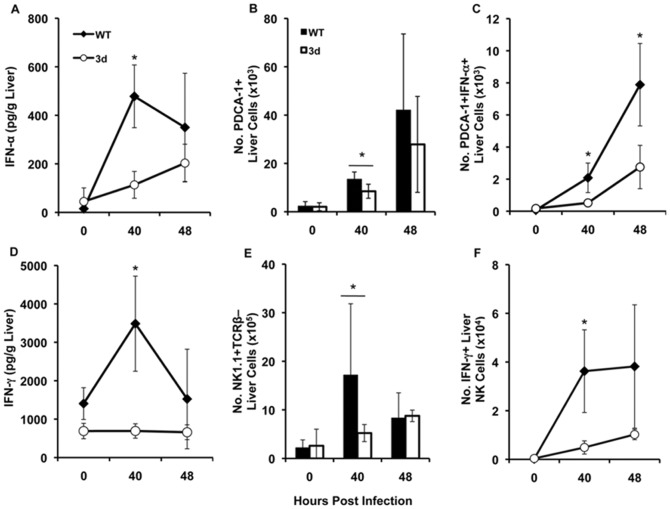
Effect of the 3d mutation on liver cytokine production and innate effector cell accumulation during MCMV infection. (A and D) Liver homogenates were generated from C57BL/6 (WT) or 3d mice that were either uninfected or infected for 40 h or 48 h with MCMV. Concentration of (A) IFN-α and (D) IFN-γ in liver homogenates was measured by sandwich ELISA. Data are the combined results from two independent experiments and show the mean ± SD (n = 3–6 mice per group). (B, C, E, F) Liver leukocytes were prepared from C57BL/6 (WT) or 3d mice that were either uninfected or infected with MCMV for 40 h or 48 h. Leukocytes were then stained for cell surface expression of PDCA-1 or NK1.1 and TCRβ, fixed, permeabilized, and stained for intracellular IFN-α or IFN-γ, as described in Methods. Results show the total numbers of (B) PDCA-1+, (C) IFN-α+ PDCA-1+, (E) NK1.1+ TCRβ– and (F) IFN-γ+ NK1.1+ TCRβ– cells. Data are the combined results from 3–4 independent experiments (B, E) or at least two independent experiments (C, F) and show the mean ± SD (n = 3–11 mice per group for each time point tested). Asterisks denote p values≤0.04.

Having observed a reduction in the level of proinflammatory cytokines in the serum of 3d mice, cytokine responses in liver cells from 3d mice infected with MCMV were evaluated to address the impact of endosomal TLR signaling in a localized tissue site of infection. The best characterized liver cytokine responses are IFN-α and IFN-γ [20], [21], [36]. Therefore, to determine whether combined endosomal TLR signaling contributes to the production of these cytokines during MCMV infection, IFN-α and IFN-γ in liver homogenates and in individual cell populations were measured in WT and 3d mice uninfected or infected with MCMV for 40 h and 48 h. As shown in Fig. 2A, WT mice displayed four-fold higher levels of IFN-α than 3d mice at 40 h post-infection, with increased levels still evident at 48 h following infection. Since pDCs expressing the marker PDCA-1 have been shown to produce the majority of liver IFN-α at 40 h post-MCMV infection [20], this cell type was examined in WT and 3d livers during early infection. There was evidence of pDC accumulation in the livers of both WT and 3d mice (Fig. 2B). However, liver pDCs from 3d mice were impaired in their ability to express IFN-α. There were 4-fold fewer PDCA-1+ pDCs expressing intracellular IFN-α at 40 h, and 3-fold fewer at 48 h post-infection, in 3d mice as compared to WT (Fig. 2C). This trend was also reflected in the proportion of PDCA-1+ IFN-α+ pDCs at 40 h and 48 h after infection in 3d mice (0.8%±0.4% and 2%±1%) compared with WT (2%±1% and 6%±1%).

3d mice similarly demonstrated a defect in liver IFN-γ production in response to MCMV infection. In WT mice, IFN-γ reached maximal levels of 3500±1200 pg/g liver at 40 h before contracting by approximately half at 48 h post-infection. In contrast, at 40 h post-infection, 3d mice induced 5-fold less IFN-γ than WT (Fig. 2D). NK cells are an important early source of IFN-γ in the liver during MCMV infection [24], [25], and accumulated at this site in both WT and 3d mice (Fig. 2E). Using intracellular cytokine staining, results shown in Fig. 2F demonstrate a 7-fold reduction in the absolute numbers of NK1.1+ TCRβ- liver cells expressing IFN-γ in 3d mice at 40 h post-infection as compared to WT. There were also fewer IFN-γ-expressing liver NK cells in 3d mice by proportion (0.8%±0.3% and 1%±0.4%) when compared to WT (6%±2% and 4%±0.4%) at 40 h and 48 h, respectively. Together, these results demonstrate that, in addition to an effect on systemic cytokine production, combined endosomal TLR signals can affect the expression of critical proinflammatory cytokines in the liver during MCMV infection.

### Liver T cell responses are not impaired in 3d mice during MCMV infection

**Figure 3:**
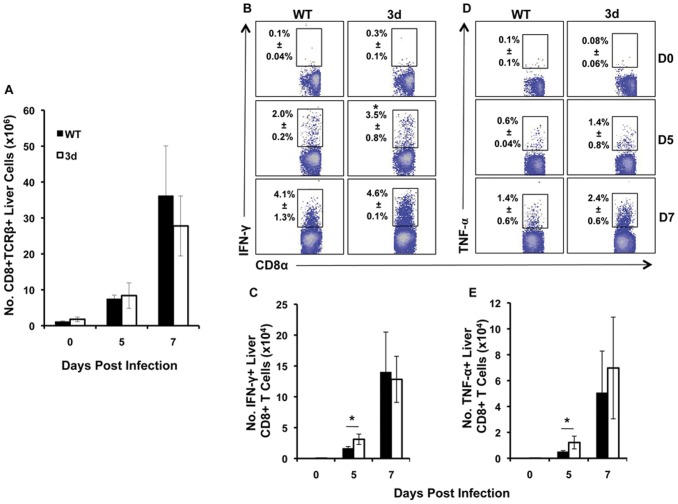
Effect of the 3d mutation on CD8+ T cell accumulation and MCMV-specific cytokine responses. Liver leukocytes were prepared from C57BL/6 (WT) and 3d mice that were either uninfected or infected with MCMV for 5 or 7 days and analyzed by flow cytometry. (A) The total numbers of CD8α+ TCRβ+ cells per liver are shown. To quantitate intracellular expression of cytokines in activated CD8+ T cells, prepared liver leukocytes were cultured with M45 viral peptide, and then harvested and stained for surface expression of CD8α and TCRβ followed by intracellular IFN-γ or TNF-α as described in Methods. (B and C) Results show the proportion and total numbers of CD8α+ TCRβ+ IFN-γ+ and (D and E) CD8α+ TCRβ+ TNF-α+ (D and E) cells. Data are representative of six independent experiments and show the mean ± SD (n = 3 mice per group). Asterisks denote p values≤0.02.

The innate immune response is important both in establishing early control of virus replication and in coordinating downstream adaptive responses. Following MCMV infection, virus-specific CD8+ T cells are recruited to the liver within 5 days and control viral replication at this site through release of cytotoxic molecules and production of cytokines such as IFN-γ and TNF-α [26], [27]. Given the abated liver cytokine responses observed in 3d mice, the effect of endosomal TLR signaling on liver CD8+ T cell responses was examined at late time points during acute MCMV infection. The results shown in Fig. 3A demonstrate comparable absolute numbers of CD8+ T cells in WT and 3d mice at days 5 and 7 post-MCMV infection, a trend that was also reflected in proportion (data not shown). To determine whether CD8+ T cells in 3d mice were properly activated against MCMV infection, intracellular expression of IFN-γ and TNF-α in CD8+ T cells was examined following *ex vivo* restimulation with H-2D^b^ M45 viral peptide, an immunodominant epitope of MCMV [29]. CD8+ T cells from 3d mice expressed these two cytokines at day 5 and day 7 post-MCMV infection by proportion and absolute numbers at levels that were comparable or slightly increased over WT (Fig. 3, B–E). As an indication of degranulation, surface expression of CD107a was also examined on liver CD8+ T cells from mice infected with MCMV; however, no differences in CD107a expression were detected between 3d and WT mice (data not shown). These results suggest that MCMV-specific CD8+ T cell responses in the liver are not compromised in the absence of endosomal TLR signals.

### Increased sensitivity to virus-induced liver disease in 3d mice during MCMV infection

**Figure 4:**
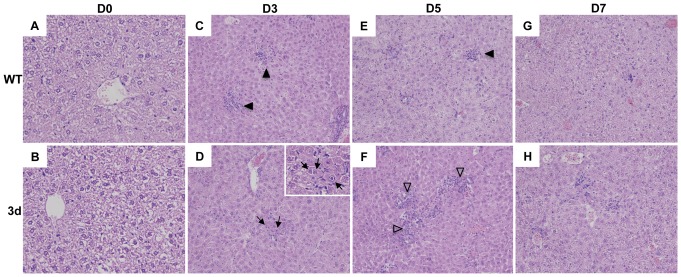
Characterization of virus-induced liver disease in 3d mice. Livers were harvested from C57BL/6 (WT) (A, C, E, G) or 3d mice (B, D, F, H) that were either uninfected (A and B) or infected for 3 (C and D), 5 (E and F) or 7 (G and H) days with MCMV (5×10^4^ PFU). Paraffin-embedded blocks were sectioned, stained with H&E, and analyzed by microscopy. Images were digitally captured at the original magnification of ×10 or at ×20 (inset in D). Arrowheads in C and E indicate inflammatory foci; arrows and inset in D indicate cytomegalic inclusions; open arrowheads in F denote areas of inflammation.

Previous studies have demonstrated resolution of virus-induced liver disease after 5 days of MCMV infection in WT mice [22], [24], [27], [37]. Therefore, given the impaired inflammatory responses observed in the absence of endosomal TLR signaling, liver sections prepared from 3d and WT mice that were uninfected or infected for 3, 5, or 7 days were hematoxylin and eosin (H&E) stained to evaluate pathology. The histological appearance of liver sections from uninfected 3d and WT mice appeared comparable (Fig. 4, A and B). By day 3 post-MCMV infection, clusters of infiltrating cells or inflammatory foci, which have been shown to coincide with sites of MCMV antigen expression [23], [37], were present in WT mice and persisted through day 5 before inflammation was resolved by day 7 post-infection (Table 1, Fig. 4, C, E and G). While the inflammatory foci per area of liver were equally apparent in liver sections from 3d mice infected for 3 days (Table 1), there was an increased presence of cytomegalic inclusion bodies characteristic of MCMV-infected cells that were not readily apparent in WT mice (Fig. 4D). By day 5 post-infection, livers from 3d mice were characterized by widespread areas of inflammation compared to the more punctate foci in WT livers (Fig. 4, E and F). Moreover, the inflammatory foci per area of liver in 3d mice at day 5 post-infection were significantly more numerous and contained a greater number of nucleated cells compared to WT (Table 1). However, by day 7 post-infection, inflammation in 3d mice showed signs of resolution that were similar to WT (Table 1, Fig. 4, G and H).

**Table 1:** Quantitation of liver inflammatory foci and cell infiltrates in WT and 3d mice during MCMV infection.

	No. Foci per 50 µm^2*a*^				No. Nucleated cells per focus^a^			
Strain	Day 0	Day 3	Day 5	Day 7	Day 0	Day 3	Day 5	Day 7
**WT**	<1	40±16	32±8	8±8	0	44±12	59±12	27±3
**3d**	<1	40±8	112±24^b^	24±16	0	45±2	>60*^c^*	36±6

a Data presented represent the mean ± SD of two liver sections unless otherwise indicated.

b Number of foci per 8×50 µm^2^ areas in day 5 infected 3d livers was significantly higher than WT at the same infection time point, p≤0.04.

C Nucleated cells from large areas of inflammation contained >60 nucleated cells in less defined foci, and do not include a calculation of SD.

**Figure 5:**
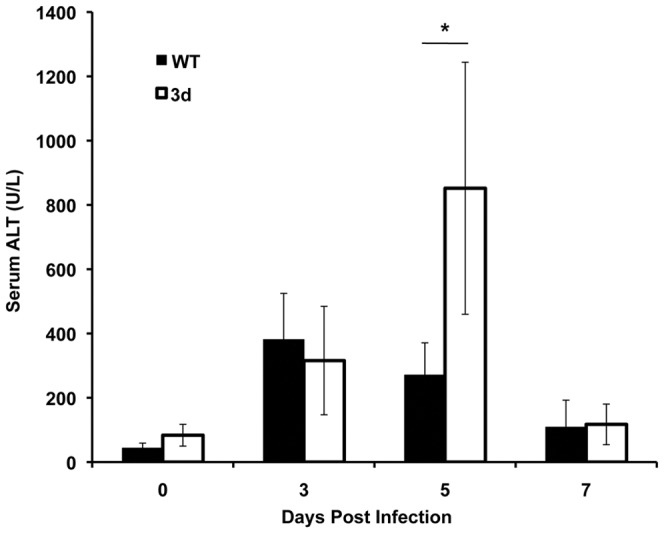
Assessment of virus-induced liver disease in 3d mice. Serum samples were collected from C57BL/6 (WT) or 3d mice that were either uninfected or infected for 3, 5, or 7 days with MCMV. Circulating levels of ALT were measured as described in Methods. Data are the combined results from three independent experiments and show the mean ± SD (n = 6–8 mice per group). Asterisks denote p values≤0.003.

To further evaluate the effects of endosomal TLR responses on overall liver function, the liver enzyme alanine aminotransferase (ALT) was measured in serum samples from WT and 3d mice that were uninfected or infected with MCMV for 3, 5 or 7 days. Uninfected mice had comparable baseline levels of systemic ALT (Fig. 5). By day 3 of infection, similar elevations in ALT levels were detected in both groups. In contrast, by day 5 post-infection, the levels of systemic ALT in 3d mice reached values of 900±400 U/L and were three-fold higher than the ALT levels measured in WT mice (270±100 U/L; Fig. 5), indicating augmented liver disease. In contrast, by day 7 post-infection, 3d mice demonstrated a sharp decline in systemic ALT levels to values of 117±63 U/L, which were comparable to ALT levels exhibited in WT mice (108±85 U/L). Thus, at this time point of acute infection, both groups of mice exhibited ALT levels near baseline levels, consistent with the resolution of virus-induced liver pathology observed in 3d and WT mice (Fig. 4, G and H; Fig. 5). Taken together, these results suggest that in the absence of endosomal TLR signals, there is pronounced liver inflammation accompanied by a transient increase in liver damage during acute MCMV infection.

### Impairment of viral clearance in 3d mice

**Figure 6:**
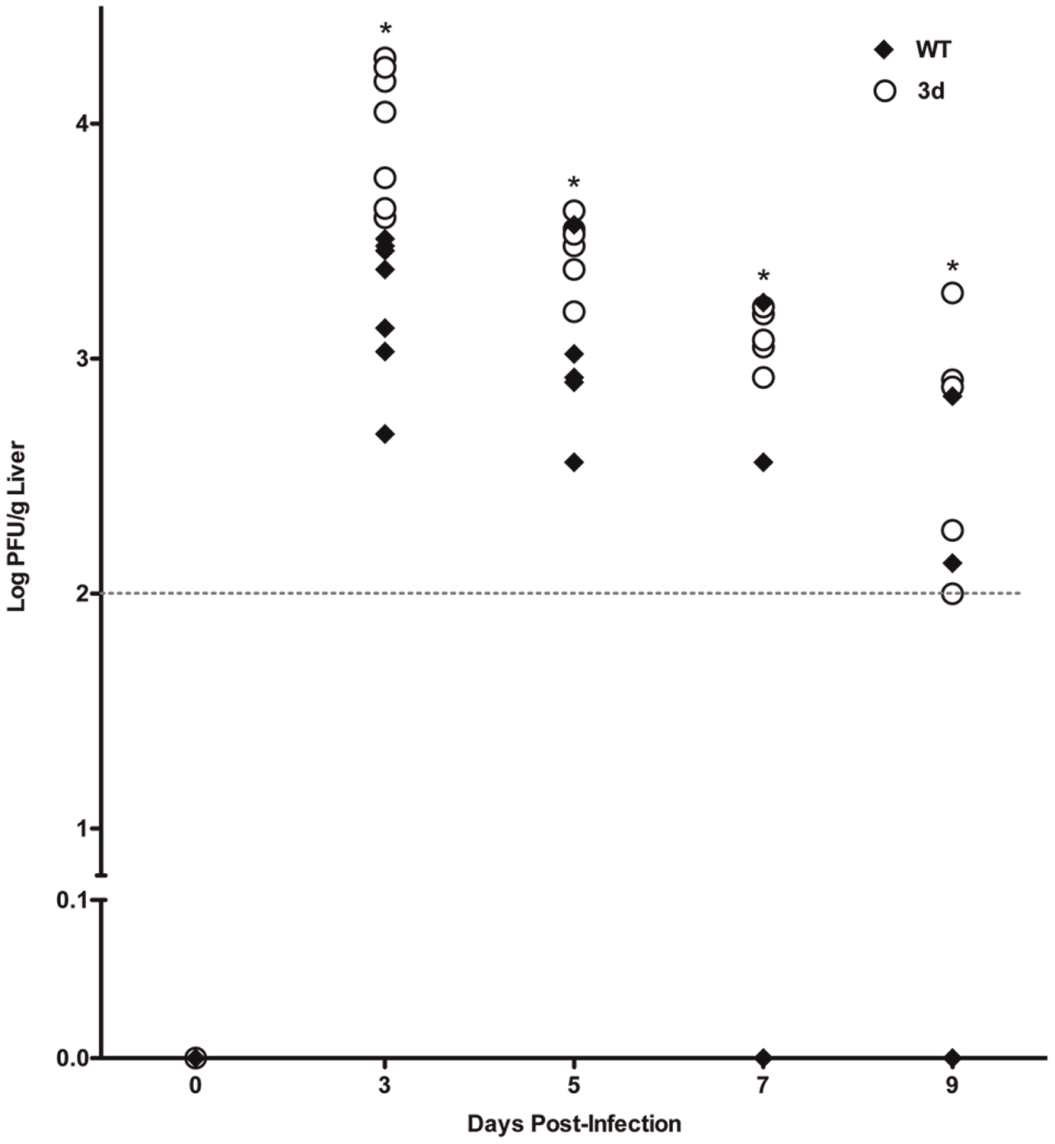
Effect of the 3d mutation on viral clearance. Livers were harvested from C57BL/6 (WT) or 3d mice that were either uninfected or infected for 3, 5, 7 or 9 days with MCMV (5×10^4^ PFU). Viral titers were determined using a standard plaque assay. The level of detection of the plaque assay is 2 log PFU/g liver (dashed line). Each data point represents an individual WT (filled diamonds) or 3d mouse (open circles). Data from days 0, 3, 5, and 7 are the combined results of three independent experiments (n = 4–7 mice per group). In data from day 9, n = 5 mice per group. Asterisks denote a significant difference between WT and 3d mean PFU/g liver (p values≤0.04).

Given the diminished early cytokine responses and enhanced liver disease observed in 3d mice, the contribution of endosomal TLR responses to control of virus replication in the liver was assessed in WT and 3d mice infected with MCMV. Compared with those in WT mice, viral titers were elevated by 1 log on day 3 of infection in 3d mice, and remained significantly higher at day 5 post-infection when compared to WT (Fig. 6). Ultimately, while WT livers showed evidence of viral clearance at days 7 and 9 post-infection, 3d liver virus titers remained approximately 2 logs higher at these times of infection as compared to WT mice. Thus, endosomal TLR signaling contributes to the control of MCMV replication in the liver during acute infection.

## Discussion

The aim of these studies was to identify the TLR signaling pathways required for the innate recognition of virus infection in the liver, a common target organ of many viruses that significantly contributes to innate immune defenses [38]–[40]. Moreover, the liver contains various innate immune cells that express TLRs [41], [42]; however, the role of TLRs in host defense against infection at this site remains largely unclear. Because responses in the liver do not appear dependent on individual TLRs [6], [20], [32], we utilized 3d mice, which lack endosomal TLR3, TLR7 and TLR9 signaling due to a mutation in the endoplasmic reticulum-resident protein UNC93B1 [34], [35], to address the contribution of endosomal TLRs in liver antiviral defenses against acute MCMV infection. The results demonstrated impaired production of proinflammatory cytokines by NK cells and pDCs in livers from MCMV-infected 3d mice. Additionally, 3d mice had elevated viral titers in the liver that coincided with transient but exacerbated liver disease, although virus-specific CD8+ T cell responses were not affected. Interestingly, a previous study demonstrated that TLR3 was not required for the generation of adaptive antiviral responses to MCMV [43], although there is evidence that TLR3 signaling contributes in part to the early control of MCMV infection by the systemic induction of type I IFN [6]. Other studies have implicated a synergistic role for TLR7 and TLR9 in promoting MCMV recognition and immune defense in the spleen [33]. The impaired liver responses observed in 3d mice that were not apparent in TLR9 or TLR7-deficient mice [20] suggests that a level of redundancy unique to innate immunity is in place within infected tissue sites to rapidly respond to viral infection. Overall, these studies advance our understanding of the process of viral recognition in the complex liver environment and suggest that UNC93B1 is a critical intermediate factor in innate virus sensing activated by MCMV.

Previous studies have demonstrated impaired serum cytokine production and increased susceptibility to infection in 3d mice infected with a high lethal dose of MCMV [35]; however, our study is the first report to document the contribution of the 3d mutation to impaired MCMV defense in the liver. In agreement with the study by Tabeta *et al.* [35], our results, using a moderate dose of MCMV, demonstrated a diminished serum cytokine response (Fig. 1), reduced splenic IFN-α production and elevated viral titers in the spleen (data not shown). These results were not unexpected given the known role for the nucleic acid-sensing TLRs in MCMV recognition and the subsequent production of proinflammatory cytokines and type I IFN by splenic pDCs [19], [31], [33], [44]. Production of type I IFN is a critical early step in antiviral defense, and we further demonstrated diminished levels of liver IFN-α in MCMV-infected 3d mice. This defect was in part due to an impaired ability of liver pDCs to express this cytokine and is consistent with previous reports identifying pDCs as an important early source of IFN-α in response to TLR7 and TLR9 ligands [10], [20], [31], [32], [33], [45]–[47]. Altogether, these results concur with previous studies indicating that pDCs are the predominant leukocyte producer of type I IFN in the liver during early MCMV infection [20], and demonstrate that production of these cytokines by pDCs is influenced by the 3d mutation.

Despite impairments in liver IFN-α production in 3d mice, it is notable that this response was not totally abrogated. This suggests the presence of alternative pathways to type I IFN production in the liver, and may be the reason that IFNα/βR-deficient mice die by day 5 in response to infection with a moderate dose of MCMV, as reported previously [36], while 3d mice do not. Studies have also demonstrated production of type I interferon from cells other than pDCs at 44–48 hours following MCMV infection [32], [48], [49]. In addition, the liver contains a variety of parenchymal and non-parenchymal cells that express TLRs and are capable of type I IFN production [38], [40]–[42]. There may also be TLR-independent pathways leading to the production of these cytokines in response to MCMV, including cytoplasmic RNA- and DNA-sensing receptors, as have been demonstrated with other virus models but have yet to be examined in the context of MCMV infection [1]–[4], [50]–[55].

It has been established that NK cell inflammatory responses and production of IFN-γ are essential to defense against MCMV in the liver [23]–[25]. In 3d mice, NK cell production of IFN-γ in the liver was severely impaired during early MCMV infection and likely contributed to increased viral burden and liver pathology. The reduced levels of IFN-γ in NK cells may reflect the deficiency of serum IL-12 seen in 3d mice. These results concur with previous reports that type I IFN regulates IL-12 production by conventional DCs and consequently the production of IFN-γ by NK cells [19], [48], [56]. The defect in liver cytokine production in 3d mice is reminiscent of that reported for MyD88-deficient mice. Notably, however, MyD88-deficient mice exhibited more severe liver pathology and greater elevations in viral titers when compared to WT than we observed in 3d mice [20]. Interestingly, mice deficient in TLR7 and TLR9 exhibited decreased levels of systemic IFN-α/β and increased susceptibility to MCMV infection [33]. Taken together, these observations further support the notion that the liver possesses compensatory mechanisms to combat viral infection in the combined absence of endosomal TLR signals.

Accordingly, despite the early effects of endosomal TLR deficiency, 3d mice were able to mount robust CD8+ T cell cytokine responses. It should be noted that the 3d defect has previously been shown to impair exogenous antigen presentation, including cross presentation, which has a reported role in priming CD8+ T cell responses during MCMV infection [35]. However, we detected no overt defect in CD8+ T cell responses within the first seven days of MCMV infection in the liver. Further, examination of activation markers suggested that a similar proportion of CD8+ T cells from 3d mice were more highly activated when compared to WT (data not shown). Several studies have demonstrated the contribution of activated virus-specific CD8+ T cells to effective hepatic immunity against MCMV infection [26]–[28]. In addition, the normal development of adaptive responses despite impaired innate responses is well documented during MCMV infection. Studies have shown that reduced levels of type I IFN do not affect the accumulation or activation of antigen-specific CD8+ T cells in response to low or moderate MCMV inoculums [48]. Likewise, while IL-12 is critical in inducing NK cell IFN-γ expression, T cell responses can occur in an IL-12-independent manner [17], [18], [57]–[59]. NK cells have the potential to negatively regulate CD8+ T cell responses during MCMV infection [60]–[62]; thus, it is probable that impaired NK cell function in the absence of endosomal TLR signals contributes to inflated T cell responses. The presence of increased virus in the liver may also contribute to the robust T cell recruitment and cytokine production observed at late infection time points in 3d mice. Interestingly, although the severity of viral liver pathology was more pronounced in 3d mice, inflammation and liver injury subsided late in infection. These observations suggest that CD8+ T cells in the liver can respond to limited amounts of type I interferon for activation in the presence of compromised innate responses, emphasizing the prevalence of compensatory mechanisms in place within the liver to deal with infection and promote adaptive immunity. In contrast, IFN-α/βR deficiency negatively impacts innate inflammatory responses and resistance to MCMV infection in the liver [21], [24], [36].

In conclusion, this study indicates that UNC93B1, which is essential for combined endosomal TLR signaling, contributes to development of effective innate immune responses to an acute virus infection in the liver. Our results show that this contribution involves modulation of early innate proinflammatory cytokine production from liver pDCs and NK cells and subsequent control of MCMV replication and pathology before activation of an adaptive immune response. Altogether, these results highlight a process of virus recognition with multiple pathways in place to promote host resistance to infection in the liver microenvironment.

## Methods

### Mice

Pathogen-free C57BL/6J mice were obtained from the Jackson Laboratory (Bar Harbor, ME). C57BL/6 *Unc93b1^3d/3d^* mice were a kind gift from Dr. Bruce Beutler (The Scripps Research Institute, La Jolla, CA) and were generated as described [35]. C57BL/6J mice were housed and *UNC93b1^3d/3d^* mice were bred in pathogen-free mouse facilities at Brown University. Male and female mice aged 8–10 weeks were used in experiments. This study was carried out in strict accordance with the recommendations in the Guide for the Care and Use of Laboratory Animals of the National Institutes of Health. All animal work was approved by the Brown University Institutional Animal Care and Use Committee (Protocol Number: 0909082 and 0903035).

### Virus Infection and viral titer determination

MCMV Smith strain was used in all experiments. This strain was prepared as a salivary gland-passaged stock from CD1 mice. Moderate dose infection (5×10^4^ PFU per mouse) was initiated on day 0 by intraperitoneal injection. In vivo responses were measured at indicated time points. For infectious viral titer quantification, organs were weighed, homogenized in cold supplemented DMEM (Invitrogen Life Technologies) and supernatants were collected following centrifugation. Serially diluted samples were used to inoculate confluent monolayers of bone marrow stromal cells (ATCC M2-10B4) in 24-well tissue culture plates and incubated for one hour at 37°C, 5% CO_2_. Following incubation, inoculums were removed and monolayers were overlaid with a 1×DMEM/0.5% low-melt agarose solution. Cells were incubated for 7 days at 37°C, 5% CO_2_, then fixed in 10% buffered formalin and stained with crystal violet. Plaques were counted to determine viral titer as previously described [22], [24], [26].

### Sample preparation

Liver leukocytes were prepared as previously described [23]–[25]. Briefly, following mechanical dissociation of tissue, red blood cells were removed by lysis with ammonium chloride and leukocytes separated by Percoll density gradient. To generate homogenates for cytokine analysis, the liver caudate lobe was homogenized in RPMI 1640 (Invitrogen Life Technologies) and supernatants collected following centrifugation. Serum was isolated from whole blood by centrifugation in the presence of heparin and stored at −80°C until further use in cytokine analyses or ALT assays.

### Cytokine analysis

Liver homogenates and serum were tested for cytokines by standard sandwich ELISA. IFN-γ, IL-12p70 and TNF-α were assayed by DuoSet (R&D Systems). IFN-α was measured by VeriKine mouse ELISA kit (R&D Systems). Limits of detection were 15 pg/mL for DuoSets and 12.5 pg/mL for VeriKine ELISA kits.

### Flow cytometric analysis

The following fluorochrome-conjugated mAbs were used in flow cytometric analyses: NK1.1-PE and TCRβ-APC to distinguish NK cells; CD8α-PECy7 and TCRβ-FITC to distinguish CD8+ T cells; and PDCA-1-APC (Miltenyi Biotec) to identify pDCs. Prior to surface staining, cells were incubated with anti-CD16/CD32 mAb to block nonspecific binding of Abs to Fcγ III/II receptors (clone 2.4G2). Unless otherwise noted, antibodies were obtained from BD Biosciences or eBioscience. For intracellular staining of cytokines, cells were treated with Brefeldin A (eBioscience) for 4 hours at 37°C, 5% CO_2_ and permeabilized prior to labeling with IFN-α-FITC (R&D Systems), IFN-γ-PE, or TNF-α-APC (BD Biosciences). When indicated, leukocytes were stimulated for 5 hours with 100 ng/mL MCMV M45 peptide in addition to Brefeldin A treatment. Samples were acquired using a FACSCalibur and analyzed with BD Cell Quest software. For analysis, viable cells were gated by FSC and SSC. Isotype controls were used to set positive analysis gates.

### Liver histology and serum ALT analysis

Portions of the median liver lobes were isolated, fixed in 10% neutral buffered formalin, and paraffin embedded. Tissue sections (5 µm) were stained with H&E and analyzed microscopically. Images shown were photographed at the indicated magnification with a DP70 digital camera and software (Optical Analysis Corporation). Inflammatory foci, defined as discrete clusters containing between 6–60 nucleated cells, were quantitated as described previously [23]. In brief, inflammatory foci were identified, at a magnification of 200, as clusters of cells in totals of 8×50 µm^2^ areas per representative tissue. In some cases, liver sections had larger areas of inflammation with >60 cells with less defined foci, and are indicated as such. Numbers of nucleated cells per inflammatory foci were counted in 20 individual foci per representative tissue at a magnification of 400. Liver alanine aminotransferase (ALT) was measured in serum samples by Marshfield Labs (Marshfield, WI).

### Statistical analysis

Student's *t* test was used to determine statistical significance of experimental results when indicated (p≤0.05).
